# Kidney involvement in VEXAS syndrome: insights from a rare case of secondary amyloidosis and systematic review of renal biopsy-confirmed reports

**DOI:** 10.1007/s10067-025-07506-9

**Published:** 2025-05-30

**Authors:** Gabriel Ştefan, Andreea Niculescu, Simona Cinca, Corina Chiriac, Adrian Zugravu, Pavel Cristina, Razvan Adrian Ionescu, Valer Mihai Pompilian, Nicoleta Petre, Cristina Căpusa, Simona Stancu

**Affiliations:** 1 University of Medicine and Pharmacy “Carol Davila”, Bucharest, Romania; 2 “Dr. Carol Davila” Teaching Hospital of Nephrology, Bucharest, Romania; 3 Internal Medicine Clinic, “Colentina” Hospital, Bucharest, Romania

**Keywords:** Kidney biopsy, Secondary amyloidosis, UBA1 gene, VEXAS syndrome

## Abstract

VEXAS syndrome is a recently identified autoinflammatory disorder caused by somatic mutations in the UBA1 gene, leading to systemic inflammation and hematologic abnormalities. While its renal involvement remains poorly understood, reported cases suggest a diverse spectrum of kidney pathology. We present a 69-year-old male with a history of systemic inflammation who developed nephrotic syndrome and worsening kidney function. His disease course included recurrent fevers, pulmonary infiltrates, cutaneous vasculitis, and hematologic abnormalities. A kidney biopsy revealed secondary amyloidosis, indicating chronic inflammation. Genetic testing confirmed a UBA1 mutation (c.121A > G, p.Met41Val), establishing the diagnosis of VEXAS syndrome. Despite treatment with corticosteroids, cyclosporine, and interleukin-1 blockade, the patient deteriorated and ultimately succumbed to septic shock. A systematic review of biopsy-confirmed renal involvement in VEXAS syndrome identified 23 cases, with interstitial nephritis as the most frequent histopathologic finding, followed by vasculitis, IgA nephropathy, minimal change disease, and amyloidosis. Treatment responses varied, with limited efficacy of immunosuppressive therapies. This case highlights the under-recognized renal manifestations of VEXAS syndrome, demonstrating its diagnostic challenges and the need for heightened clinical suspicion. Further research is required to define optimal management strategies and improve patient outcomes.

**Supplementary Information:**

The online version contains supplementary material available at 10.1007/s10067-025-07506-9.

## Introduction

In 2020, researchers at the National Institutes of Health identified a somatic mutation in the UBA1 gene as the cause of a subset of late-onset, treatment-refractory inflammatory syndrome with hematologic abnormalities [1]. This condition, termed VEXAS syndrome (Vacuoles, E1 enzyme, X-linked, Autoinflammatory, Somatic), is characterized by mutations at codon 41 of the UBA1 gene on the X chromosome, leading to impaired ubiquitin-activating enzyme 1 function [1]. The resulting disruption in protein regulation and inflammatory processes manifests in a broad array of systemic symptoms, including recurrent fever, skin lesions (e.g., neutrophilic dermatosis, leukocytoclastic vasculitis), auricular and nasal chondritis, pulmonary infiltrates, macrocytic anemia, and thrombocytopenia [2, 3]. Bone marrow examination consistently reveals vacuolization of myeloid and erythroid precursors, a hallmark finding of VEXAS [3].

The clinical presentation of VEXAS syndrome often mimics conditions such as relapsing polychondritis, myelodysplastic syndrome, polyarteritis nodosa, and giant cell arteritis, leading to frequent misdiagnosis and delays in treatment [3]. The overlapping symptoms with these well-established inflammatory and hematologic disorders complicate early identification, underscoring the importance of genetic testing in suspected cases [3].

Renal involvement in VEXAS syndrome, though reported in only a subset of cases, remains an area of limited understanding. Existing reports describe a range of kidney pathologies, including tubulointerstitial nephritis, anti-MPO vasculitis, IgA vasculitis, and AA amyloidosis, but detailed data on associated clinical findings and management strategies are sparse [4–6]. This review focuses on a clinical case of kidney secondary amyloidosis in VEXAS syndrome and systematically examines the renal involvement in VEXAS, including histopathologic features and therapeutic approaches observed in patients who underwent kidney biopsy.

## Clinical case

**Fig. 1:**
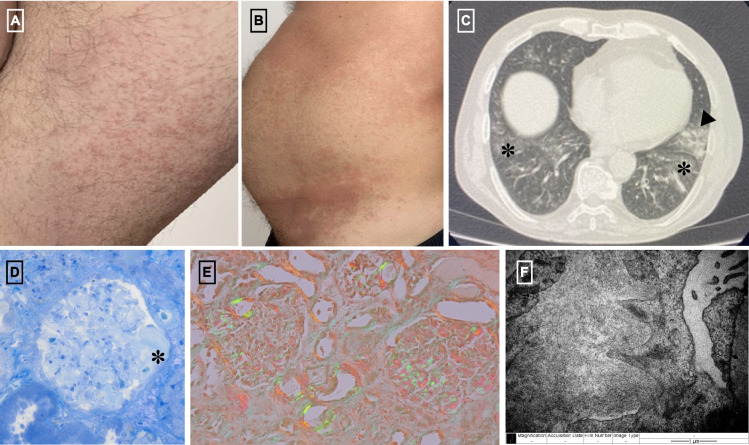
**A** Erythematous rash on the thigh; **B** Erythematous rash on the abdomen with a tendency to coalesce; **C** Thoracic computed tomography findings of bilateral diffuse ground-glass opacifications (asterisk) and left upper lobe consolidation (arrowhead); **D**, **E** In optic microscopy the glomeruli showed amorphous deposits in the mesangial spaces (asterisk, toluidine blue coloration) and segmentally along the capillary walls, exhibiting green birefringence under polarized light with Congo red staining. **F** Transmission electron microscopy image shows the presence of frequent organized deposits with fibrillar ultrastructure, approximately 12 nm in diameter, arranged randomly and focally in a spiculated pattern, located both in the mesangial spaces and segmentally along the capillary walls

A 69-year-old male was referred to our clinic in March 2024 for evaluation of nephrotic syndrome after laboratory findings showed significant proteinuria (3.1 g/day), hypoalbuminemia (2.2 g/dL), and mildly impaired kidney function with a serum creatinine level of 1.23 mg/dL and an estimated glomerular filtration rate (eGFR) of 60 mL/min (CKD-EPI 2009). His medical history included an autoinflammatory systemic disease that began four years earlier with recurrent fever, transient generalized rash, and dyspnea persisting despite empiric antibiotic therapy. Initial investigations revealed marked systemic inflammation (C-reactive protein [CRP]: 182 mg/L) and thoracic computed tomography (CT) findings of bilateral diffuse ground-glass opacifications and left upper lobe consolidation (Fig. 1C). The clinical presentation was consistent with bronchopneumonia, though no pathogen was identified. Despite improvement with broad-spectrum antibiotics, his symptoms recurred within weeks, along with erythematous rash on the dorsal hands, thighs and abdomen (Fig. 1A and B). A rheumatologic evaluation suggested a systemic inflammatory disease, but no specific diagnostic criteria were met. Treatment with methylprednisolone and azathioprine (100 mg/day) resulted in symptom improvement and a reduction in CRP levels.

During the COVID-19 pandemic, the patient discontinued follow-up and ceased azathioprine, self-adjusting corticosteroid doses based on symptom severity. In February 2023, he presented with persistent emesis. Laboratory work showed a CRP of 300 mg/L, a normal white blood cell count, and abdominal CT findings suggestive of mesenteric panniculitis. His condition worsened with new-onset aphasia and tonic–clonic seizures. Cerebrospinal fluid (CSF) analysis showed elevated protein (2.5 g/dL), reduced glucose (37 mg/dL), and a white blood cell count of 494/µL, consistent with bacterial meningoencephalitis, though no pathogen was identified. He responded well to antibiotics and corticosteroids.

Following recovery, in light of persistently elevated serum ferritin levels ranging from 2 000 to 3 500 ng/mL, adult-onset Still’s disease was suspected. Corticosteroids were continued, and azathioprine was reinitiated; however, CRP levels remained elevated in the following months. In August 2023, he developed a pulmonary embolism and was started on apixaban.

Since November 2023, laboratory work revealed proteinuria and hypoalbuminemia, raising suspicion for nephrotic syndrome. These findings prompted his referral to our clinic for further evaluation.

On examination, the patient appeared chronically ill, with a temporal temperature of 36.2 °C, blood pressure of 80/50 mmHg, and a pulse of 78 beats per minute. He was alert, with normal respiratory rate and oxygen saturation of 94% on ambient air. The patient was pale, exhibited hypotonia, hypokinesia, and hyposthenia, but showed no signs of edema.

The initial laboratory results, summarized in Table 1 (Annex), revealed mild normochromic normocytic anemia, evidence of systemic inflammation, elevated ferritin levels, and hypokalemia. Besides nephrotic syndrome, the patient exhibited worsening kidney function compared to prior assessments, with a serum creatinine level of 2.08 mg/dL. Urinary sediment analysis showed no hematuria.

The kidney biopsy revealed amorphous mesangial deposits on light microscopy (Fig. 1D), which were Congo red-positive and exhibited green birefringence under polarized light (Fig. 1E). Immunofluorescence demonstrated no light chain restriction. Electron microscopy identified randomly distributed fibrils localized segmentally within the capillary walls and mesangium (Fig. 1F). These findings were consistent with a diagnosis of renal secondary amyloidosis.

Given the patient's advanced age at the onset of systemic autoinflammatory disease, male sex, and history of multi-organ involvement—pulmonary (recurrent pneumonia without an identifiable infectious cause), cerebral (non-infectious meningoencephalitis), intestinal (mesenteric panniculitis), hematologic (bicytopenia with moderate macrocytic anemia and thrombocytopenia), and cutaneous (purpuric and necrotizing rashes, along with a non-pruritic abdominal rash)—we considered the possibility of VEXAS syndrome. Kidney biopsy findings, consistent with secondary amyloidosis, further supported this hypothesis by linking chronic inflammation to organ damage. Genetic testing confirmed the diagnosis with the identification of a pathogenic UBA1 gene variant: c.121 A > G, p.Met41 Val.

The patient was initially treated with empiric antibiotics (vancomycin and meropenem adjusted for kidney function), which were discontinued after negative cultures. Hypotension, attributed to underfill nephrotic syndrome, was managed with human albumin. Corticosteroids were continued, and cyclosporine was initiated and well tolerated, though CRP levels remained persistently elevated. Due to a lack of clinical improvement over 14 days, he was transferred to the rheumatology department and started on an interleukin-1 (IL1) receptor antagonist (Anakinra, 100 mg/day). However, his condition rapidly deteriorated, and within six days, he developed septic shock with no identified pathogen, although high procalcitonin levels suggested an underlying infection. Despite intensive care measures, he became hemodynamically unstable and ultimately succumbed to cardiac arrest.

## Search strategy

The search strategy for the systematic review was conducted in PubMed/MEDLINE to identify studies on kidney involvement in VEXAS syndrome published between January 2020 and December 2024. The search terms included combinations of keywords such as "VEXAS syndrome," "UBA1 mutation," "renal involvement," "kidney biopsy," "secondary amyloidosis," "interstitial nephritis," "ANCA," and "vasculitis." Filters were applied to include only articles published in English and only studies that reported kidney biopsy findings. The search yielded ten relevant studies (Table 1), including case reports and cohort studies, which were analyzed for clinical presentations, histopathologic findings, therapeutic interventions, and patient outcomes.

**Table 1:** Overview of previous reports of VEXAS patients with kidney biopsy

Country, Type of report	Sex, Age (years)	UBA1 mutation	Syndrome	eGFR (mL/min)	P-uria (g/d)	Kidney biopsy result	Treatment	ESKD	Death
USA, Cohort study [4] N = 20 AKI/6 KB	M, 70	p. Met41Thr (60%) p. Met41Val (20%) p. Met41Leu (15%)	AKI	NA	NA	6 KB performed: AIN, no signs of active GN; Patterns of injury described: • Plasma cell-rich AIN with increased IgG4 positive plasma cells (3 cases); • AIN with neutrophil predominance (1 case); • Acute tubular injury without interstitial inflammation (1 case); • All cases had mild interstitial fibrosis and tubular atrophy (0–20%)	Glucocorticoids; Tocilizumab (anti-IL6R) 9 cases; Canakinumab (anti-IL-β) 2 cases; Hypomethylating agents (azacitidine 3 cases, decitabine 2 cases); JAK inhibitors 4 cases; Allogenic hematopoietic stem cell transplantation 2 cases	No	3 cases
France, Case report [12, 20]	M, 61	p. Met41Thr	CKD	110	0.12	IgA nephropathy	Glucocorticoids; Anti-IL17 therapy	No	Yes
France, Case report [12, 21]	M, 72	c. 118-1G > C Splice mutation	AKI	NA	1.8	AIN	Glucocorticoids; JAK inhibitors; Anti-IL1 therapy	Yes	No
France, Case series [12]	M, 69	p. Met41Leu	AKI	35	0.7	AIN	Glucocorticoids; JAK inhibitors; MTX	No	No
France, Case series [12]	M, 69	p. Met41Thr	Nephrotic synd	90	5.6	AIN and Minimal change disease	Glucocorticoids; JAK inhibitors; Anti-IL6R therapy; Anti-TNF therapy	No	No
France, Case series [12]	M, 73	p. Met41Val	AKI	NA	6.3	Pauci-immune crescentic glomerulonephritis	Glucocorticoids; JAK inhibitors; Anti-IL6R therapy	No	No
France, Case series [12]	M, 67	c. 118-1G > C Splice mutation	Isolated P-uria	113	3	Minimal change disease	Glucocorticoids; MTX; Lenalidomide; MMF	No	Yes
France, Case series [12]	M, 73	p. Met41Thr	AKI	NA	1.2	Acute tubular necrosis	JAK inhibitors	No	NA
France, Case series [12]	M, 77	p. Met41Leu	CKD	29	4.3	Diabetic nephropathy	Glucocorticoids; JAK inhibitors	No	No
France, Case series [12]	M, 73	p. Met41Thr	AKI	22	0.3	Acute tubular necrosis	Glucocorticoids; JAK inhibitors; Colchicine; Anti-IL6R therapy; Anti-IL1 therapy	No	No
France, Case series [12]	M, 78	p. Met41Thr	AKI	26	3.8	AIN	Glucocorticoids	No	No
Netherlands, Case series [8]	M, 69	p. Met41Thr	CKD	NA	NA	CIN: homogeneous interstitial infiltrate with MPO-positive and CD681 myeloid cells, no eosinophils, lymphocytes, or plasma cells	Glucocorticoids; CFM; MTX; Anti-IL1 therapy; Anti-IL6R therapy	No	No
Netherlands, Case series [8]	M, 47	p. Met41Thr	AUA	NA	NA	Biopsy specimen showing no abnormalities	Glucocorticoids; Anti-IL1 therapy	No	No
Spain, Case report [19]	M, 76	p. Met41Thr	Nephritic synd	20	NA	IgA nephropathy	Glucocorticoids; MTX; Anti-IL6R therapy	No	No
USA, Case series [22]	M, 70	p. Met41Thr	AKI	NA	NA	AIN and renal peritubular capillaritis (interstitial and peritubular capillary infiltrating neutrophils with extensive karyorrhectic debris)	Glucocorticoids; RTX; Hypomethylating agents; MMF; Anti-IL6R therapy	No	Yes
Italy, Case series [13]	M, 75	p. Met41Val	Nephritic synd	NA	NA	Pauci-immune crescentic glomerulonephritis	Glucocorticoids; CFM; MMF; RTX	No	No
France, Case report [6, 12]	M, 59	p. Met41Thr	Nephrotic synd	12	9	AA Amyloidosis Positive immunostaining for serum amyloid A deposits in glomeruli, tubules, and vessels	Glucocorticoids; Anti-IL1 therapy	Yes	No
Romania, Current case report	M, 69	p. Met41Val	Nephrotic synd	60	31.2	AA Amyloidosis	Glucocorticoids; Cyclosporine; Anti-IL1 therapy	No	Yes

*AIN* acute interstitial nephritis, *AKI* acute kidney injury, *AUA* asymptomatic urinary abnormalities, *eGFR* estimated glomerular filtration rate, *CFM* cyclophosphamide, *CIN* chronic interstitial nephritis, *CKD* chronic kidney disease, *ESKD* end stage kidney disease, *GN* glomerulonephritis, *IgA* immunoglobulin A, *IL* interleukin, *KB* kidney biopsy, *M* male, *MTX* methotrexate, *MMF* mycophenolate mofetil, *MPO* myeloperoxidase, *NA* not assessed, *P-uria* proteinuria, *R* receptor, *RTX* rituximab, *synd* syndrome, *USA* United States of America

## Discussion

Since the discovery of VEXAS syndrome, numerous cases, case series, and cohorts have been reported, but kidney involvement remains under-recognized despite a reported prevalence of 9.5% to 25% [4, 7]. The systematic review included 23 biopsy-confirmed cases of kidney involvement in VEXAS syndrome (Table 1). The most frequent clinical presentation was acute kidney injury (AKI), observed in 14 cases, followed by nephrotic syndrome (5 cases) and chronic kidney disease (4 cases). The most common histopathologic finding was acute interstitial nephritis, reported in 10 cases, often with plasma cell-rich infiltrates or neutrophil predominance. Other findings included pauci-immune crescentic glomerulonephritis, IgA nephropathy, minimal change disease and AA amyloidosis (Table 1).

The most frequently reported UBA1 mutation was p.Met41 Thr, accounting for approximately 60% of cases, followed by p.Met41 Val (20%) and p.Met41Leu (15%). Less common mutations, such as splice site variants (e.g., c.118-1G > C), were also reported in two cases. Treatment strategies varied and included glucocorticoids, Janus kinase (JAK) inhibitors, and anti-IL1 or anti-IL6 receptors therapies. Two cases progressed to end-stage kidney disease, and six deaths were reported, reflecting the severity and variability of kidney involvement in VEXAS syndrome.

Our case represents the second reported instance of kidney involvement due to secondary amyloidosis associated with VEXAS syndrome. Both cases highlight the significant diagnostic and therapeutic challenges posed by VEXAS syndrome-related AA amyloidosis, yet they differ notably in clinical presentation and outcomes. Both involved elderly males with chronic systemic inflammation, nephrotic syndrome secondary to AA amyloidosis confirmed by Congo red staining, and pathogenic UBA1 mutations (our case: c.121 A > G; reported case: c.122 T > C). Both cases were refractory to standard therapies, requiring corticosteroids and IL-1 inhibition [6].

Key differences include disease trajectory and severity. Our patient had moderate renal impairment initially (serum creatinine 1.23 mg/dL, proteinuria 3.1 g/day) with multi-organ involvement (pulmonary, gastrointestinal, cerebral, hematologic, and cutaneous), progressing to creatinine 2.08 mg/dL before rapid deterioration and death from septic shock during anakinra therapy. The previous reported case presented with severe renal impairment (serum creatinine 4.83 mg/dL, proteinuria 9 g/g creatinine), cardiac amyloidosis confirmed by MRI, dialysis dependency, and systemic manifestations affecting the skin (maculo-papular rash, auricular chondritis), hematologic system (macrocytic anemia), and bone marrow (vacuolization of granulocytic and erythroid progenitors) [6]. Despite these complications, the patient showed partial clinical improvement with IL-1 blocking therapy but remained dependent on hemodialysis [6].

The most common histopathologic finding in VEXAS patients undergoing kidney biopsy is plasma cell-rich interstitial nephritis with increased IgG4-positive plasma cells, often requiring differentiation from IgG4-related tubulointerstitial nephritis [4]. Interstitial nephritis with a neutrophil predominance has also been observed [7–9].

While UBA1 mutations are known to occur in peripheral myeloid cells and are associated with neutrophilic dermatitis in VEXAS, their role in kidney inflammation remains uncertain [1, 10, 11]. Immunohistochemical studies have shown that interstitial inflammatory cells in VEXAS-related kidney biopsies consist of a mix of lymphocytes, plasma cells, macrophages, and neutrophils, with co-expression of myeloperoxidase and CD68 indicating the presence of immature myeloid cells [12]. Molecular analysis of kidney biopsy samples confirmed UBA1 mutations in 87.5% (7 out of 8) of cases, with variant allele frequencies ranging from 2 to 17% of total kidney DNA [12]. These findings suggest that kidney inflammation in VEXAS is likely driven, at least in part, by UBA1-mutated cells rather than being purely secondary to systemic inflammation [12]. Further research is needed to understand the functional impact of these mutations on disease progression.

Patients with VEXAS and acute kidney injury due to interstitial nephritis often experience recurrent episodes, with a median of six episodes per patient [4]. A higher age at first presentation and elevated baseline CRP levels are significantly associated with a shorter time to the first AKI event [4]. In the largest study of VEXAS patients with AKI, prednisone treatment during follow-up was effective, improving GFR and resolving urinary abnormalities [4]. However, most relapses occurred when the prednisone dose was reduced to less than 10–15 mg daily [4].

Cases of VEXAS syndrome associated with, or mimicking ANCA-associated vasculitis (AAV) have been reported [5, 13, 14]. While kidney involvement is typically observed in AAV, only 4 of the 9 reported patients with VEXAS (44%) had renal involvement. Among them, 2 exhibited kidney lesions consistent with the typical AAV pattern (pauci-immune focal necrotizing glomerulonephritis) [13], one showed cortical peritubular leukocytoclastic capillaritis without crescentic glomerulonephritis [14], and another displayed non-specific tubular involvement [5].

Clinically, VEXAS syndrome can mimic AAV, posing a diagnostic challenge due to overlapping features. It may present with a range of autoimmune and autoinflammatory conditions, with granulomatosis with polyangiitis being the most frequently reported AAV phenotype in VEXAS [5, 14]. Notably, cases often exhibit atypical features, such as negative or incongruent ANCA results, absence of typical respiratory or sinus symptoms despite pulmonary findings on imaging, and refractory symptoms requiring prolonged glucocorticoid therapy [5].

The connection between AAV and VEXAS syndrome remains unclear but may involve shared mechanisms of neutrophil activation and neutrophil extracellular trap (NET) formation. Anti-neutrophil cytoplasmic antibodies (ANCA) play a key role in activating neutrophils in AAV, while studies show that neutrophils in VEXAS syndrome also exhibit enhanced NET formation. In VEXAS, mutations disrupt protein ubiquitylation in myeloid cells, leading to endoplasmic reticulum stress and activation of the unfolded protein response (UPR) through key pathways: PERK (Protein kinase R-like endoplasmic reticulum kinase), IRE1α (Inositol-requiring enzyme 1 alpha), and ATF6 (Activating transcription factor 6) [15]. These pathways restore endoplasmic reticulum function by reducing protein synthesis (PERK), managing misfolded proteins and regulating inflammatory processes (IRE1α), and enhancing protein folding (ATF6) [3]. This stress response triggers a pro-inflammatory cascade via cytokines such as TNF, IL-1, IL-6, IL-8, and IFN-γ, contributing to NET formation and vascular inflammation (endothelitis) [3, 16, 17]. Notably, IRE1α has been identified as a key regulator of NET formation, and its inhibition may reduce immune complex–driven NETosis [18]. This connection between neutrophil activation and the UPR offers promising therapeutic opportunities for managing VEXAS syndrome with AAV-like features [5, 18].

In addition, a case of VEXAS syndrome associated with IgA vasculitis has been reported, characterized by kidney biopsy findings of IgA nephropathy, purpura caused by leucocytoclastic vasculitis, and elevated serum IgA levels [19]. Similarly, two cases of minimal change disease have been described in VEXAS patients [12]. These findings suggest potential associations rather than causality, as no direct mechanistic link has been established. It remains uncertain whether these renal pathologies are coincidental or part of a broader spectrum of kidney involvement in VEXAS syndrome.

Our case highlights the challenges of diagnosing VEXAS syndrome-related secondary amyloidosis, a rare complication identified through kidney biopsy in the context of systemic inflammation. Renal involvement in VEXAS is under-recognized, with reports indicating diverse histopathologies such as interstitial nephritis and amyloidosis. The rapid progression in our patient despite therapy illustrates the aggressive nature of the disease and underscores the need for heightened clinical suspicion and earlier intervention to manage renal complications effectively.

## Supplementary Information

Below is the link to the electronic supplementary material.

Supplementary file1 (DOCX 16 KB)

## Data Availability

The original data presented in the study are included in the article/Supplementary Material, further inquiries can be directed to the corresponding author.

## References

[1] Beck DB, Ferrada MA, Sikora KA, Ombrello AK, Collins JC, Pei W et al (2020) Somatic mutations in UBA1 and severe adult-onset autoinflammatory disease. N Engl J Med 383(27):2628–2638. 10.1056/NEJMoa2026834 33108101 10.1056/NEJMoa2026834 PMC7847551

[2] Heiblig M, Patel BA, Groarke EM, Bourbon E, Sujobert P (2021) Toward a pathophysiology inspired treatment of VEXAS syndrome. Semin Hematol 58(4):239–246. 10.1053/j.seminhematol.2021.09.001 34802546 10.1053/j.seminhematol.2021.09.001

[3] Koster MJ, Samec MJ, Warrington KJ (2023) VEXAS syndrome-a review of pathophysiology, presentation, and prognosis. J Clin Rheumatol 29(6):298–306. 10.1097/RHU.0000000000001905 36251488 10.1097/RHU.0000000000001905

[4] Kalantari K, Sullivan MM, Hernandez LPH, Bu L, Cornell LD, Nasr SH et al (2024) Acute kidney injury, an underrecognized feature of VEXAS syndrome. Rheumatology (Oxford). 10.1093/rheumatology/keae465 10.1093/rheumatology/keae465 39186250

[5] Murillo-Chavez F, Antiochos B (2024) VEXAS syndrome as a mimicker of ANCA-associated vasculitis. Rheumatol Adv Pract 8(4):rkae116. 10.1093/rap/rkae116 39411287 10.1093/rap/rkae116 PMC11479695

[6] Euvrard R, Fournier T, Georgescu D, Bourbon E, Sujobert P, Lega JC et al (2021) VEXAS syndrome-related AA amyloidosis: a case report. Rheumatology (Oxford) 61(1):e15–e16. 10.1093/rheumatology/keab683 34498034 10.1093/rheumatology/keab683

[7] Georgin-Lavialle S, Terrier B, Guedon AF, Heiblig M, Comont T, Lazaro E et al (2022) Further characterization of clinical and laboratory features in VEXAS syndrome: large-scale analysis of a multicentre case series of 116 French patients. Br J Dermatol 186(3):564–574. 10.1111/bjd.20805 34632574 10.1111/bjd.20805

[8] van der Made CI, Potjewijd J, Hoogstins A, Willems HPJ, Kwakernaak AJ, de Sevaux RGL et al (2022) Adult-onset autoinflammation caused by somatic mutations in UBA1: a dutch case series of patients with VEXAS. J Allergy Clin Immunol. 149(1):432–9 e4. 10.1016/j.jaci.2021.05.014 34048852 10.1016/j.jaci.2021.05.014

[9] Poulter JA, Collins JC, Cargo C, De Tute RM, Evans P, Ospina Cardona D et al (2021) Novel somatic mutations in UBA1 as a cause of VEXAS syndrome. Blood 137(26):3676–3681. 10.1182/blood.2020010286 33690815 10.1182/blood.2020010286 PMC8462400

[10] Lacombe V, Beucher A, Urbanski G, Le Corre Y, Cottin L, Croue A et al (2022) Distinction between clonal and paraclonal cutaneous involvements in VEXAS syndrome. Exp Hematol Oncol 11(1):6. 10.1186/s40164-022-00262-5 35172893 10.1186/s40164-022-00262-5 PMC8848791

[11] Zakine E, Schell B, Battistella M, Vignon-Pennamen MD, Chasset F, Mahevas T et al (2021) UBA1 variations in neutrophilic dermatosis skin lesions of patients with VEXAS syndrome. JAMA Dermatol 157(11):1349–1354. 10.1001/jamadermatol.2021.3344 34495287 10.1001/jamadermatol.2021.3344 PMC8427502

[12] Mathurin M, Hirsch P, Jachiet V, Hadjadj J, Le Guenno G, Heiblig M et al (2025) A clinicopathological description of kidney features in VEXAS syndrome. Kidney Int Rep 10(1):260–264. 10.1016/j.ekir.2024.10.026 39810778 10.1016/j.ekir.2024.10.026 PMC11725828

[13] Muratore F, Marvisi C, Castrignano P, Nicoli D, Farnetti E, Bonanno O et al (2022) VEXAS syndrome: a case series from a single-center cohort of italian patients with vasculitis. Arthritis Rheumatol 74(4):665–670. 10.1002/art.41992 34611997 10.1002/art.41992 PMC8957507

[14] Koster MJ, Ghaffar U, Kermani TA, Patnaik MM, Go RS, Mangaonkar AA et al (2023) Antineutrophil cytoplasmic antibody-associated vasculitis and VEXAS syndrome: comment on the article by Muratore et al. Arthritis Rheumatol. 75(8):1490–2 36716119 10.1002/art.42466

[15] Beck DB, Werner A, Kastner DL, Aksentijevich I (2022) Disorders of ubiquitylation: unchained inflammation. Nat Rev Rheumatol 18(8):435–447. 10.1038/s41584-022-00778-4 35523963 10.1038/s41584-022-00778-4 PMC9075716

[16] Vitale A, Caggiano V, Bimonte A, Caroni F, Tosi GM, Fabbiani A et al (2023) VEXAS syndrome: a new paradigm for adult-onset monogenic autoinflammatory diseases. Intern Emerg Med 18(3):711–722. 10.1007/s11739-023-03193-z 36662445 10.1007/s11739-023-03193-z PMC10082120

[17] Oo TM, Koay JTJ, Lee SF, Lee SMS, Lim XR, Fan BE (2022) Thrombosis in VEXAS syndrome. J Thromb Thrombolysis 53(4):965–970. 10.1007/s11239-021-02608-y 34817788 10.1007/s11239-021-02608-y PMC8612112

[18] Sule G, Abuaita BH, Steffes PA, Fernandes AT, Estes SK, Dobry C et al (2021) Endoplasmic reticulum stress sensor IRE1alpha propels neutrophil hyperactivity in lupus. J Clin Invest 131(7). 10.1172/JCI137866 10.1172/JCI137866 PMC8011900 33561013

[19] Pamies A, Ferras P, Bellaubi-Pallares N, Gimenez T, Raventos A, Colobran R (2022) VEXAS syndrome: relapsing polychondritis and myelodysplastic syndrome with associated immunoglobulin A vasculitis. Rheumatology (Oxford) 61(3):e69–e71. 10.1093/rheumatology/keab782 34668539 10.1093/rheumatology/keab782

[20] Magnol M, Couvaras L, Degboe Y, Delabesse E, Bulai-Livideanu C, Ruyssen-Witrand A et al (2021) VEXAS syndrome in a patient with previous spondyloarthritis with a favourable response to intravenous immunoglobulin and anti-IL17 therapy. Rheumatology (Oxford) 60(9):e314–e315. 10.1093/rheumatology/keab211 33693498 10.1093/rheumatology/keab211

[21] Ronsin C, Benard L, Mourtada A, Perrin F, Boukerroucha Z (2022) Acute tubulointerstitial nephritis revealing VEXAS syndrome. Kidney Int 101(6):1295–1297. 10.1016/j.kint.2022.03.012 35597594 10.1016/j.kint.2022.03.012

[22] Koster MJ, Kourelis T, Reichard KK, Kermani TA, Beck DB, Cardona DO et al (2021) Clinical heterogeneity of the VEXAS syndrome: a case series. Mayo Clin Proc 96(10):2653–2659. 10.1016/j.mayocp.2021.06.006 34489099 10.1016/j.mayocp.2021.06.006

