# Supplementary material for: Kidney involvement in VEXAS syndrome: insights from a rare case of secondary amyloidosis and systematic review of renal biopsy-confirmed reports
Source: Clin Rheumatol. 2025 May 30;44(7):3101–8. doi: 10.1007/s10067-025-07506-9 (PMC12234580; doi:10.1007/s10067-025-07506-9)
Supplement: Supplementary file 1 — Supplementary file1 (DOCX 16 KB) [file 10067_2025_7506_MOESM1_ESM.docx]

*Table 1: Laboratory findings during evaluation at our clinic (PR3-ANCA = Proteinase 3-anti-neutrophil cytoplasmic antibodies; MPO-ANCA=myeloperoxidase-anti-neutrophil cytoplasmic antibodies).*

| **Parameter** | **Level** | **Normal range** |
| --- | --- | --- |
| Hemoglobin (g/dL) | 10.8 | 11.5-17 |
| Mean corpuscular volume (fL) | 90.1 | 80-100 |
| White blood cell count (per mm^3^) | 6930 | 4000-10000 |
| C reactive protein (mg/L) | 50 | 0-8 |
| Ferritin (ng/mL) | 1815.9 | 15-300 |
| Transferrin (mg/dL) | 64 | 200-360 |
| Transferrin Saturation (%) | 81% | 15-45 |
| Serum albumin (g/dL) | 2.2 | 3.5-5.2 |
| Total serum protein (g/dL) | 4.4 | 6.3-8.3 |
| Serum Creatinine (mg/dL) | 2.08 | 0.55-1.05 |
| Serum Sodium (mEq/L) | 131.1 | 135-147 |
| Serum Potassium (mEq/L) | 3.1 | 3.7-5.1 |
| Serum Immunoglobulin A (g/L) | 2.25 | 0.6-4 |
| Serum Immunoglobulin G (g/L) | 4.4 | 7-16 |
| Serum Immunoglobulin M (g/L) | 0.44 | 0.4-2.3 |
| Serum free kappa light chains (mg/L) | 56.84 | 3.3-19.4 |
| Serum free lambda light chains (mg/L) | 21.85 | 5.7-26.3 |
| Complement C3 (g/L) | 0.95 | 0.9-1.8 |
| Complement C4 (g/L) | 0.24 | 0.1-0.4 |
| Rheumatoid factor (UI/mL) | <5.8 | 0-14 |
| DNA Double-Stranded Antibodies (U/mL) | 1.58 | 0-200 |
| PR3-ANCA (RU/mL) | 0.13 | 0-20 |
| MPO-ANCA (RU/mL) | 0.17 | 0-20 |
| Proteinuria (g/day) | 31.2 | absent |
